# An efficient, scarless, selection-free technology for phage engineering

**DOI:** 10.1080/15476286.2023.2270344

**Published:** 2023-10-16

**Authors:** Moran G. Goren, Tridib Mahata, Udi Qimron

**Affiliations:** Department of Clinical Microbiology and Immunology, School of Medicine, Tel Aviv University, Tel Aviv, Israel

**Keywords:** DNA engineering, Bacteriophages, pORTMAGE

## Abstract

Most recently developed phage engineering technologies are based on the CRISPR-Cas system. Here, we present a non-CRISPR-based method for genetically engineering the *Escherichia coli* phages T5, T7, P1, and λ by adapting the pORTMAGE technology, which was developed for engineering bacterial genomes. The technology comprises *E. coli* harbouring a plasmid encoding a potent recombinase and a gene transiently silencing a repair system. Oligonucleotides with the desired phage mutation are electroporated into *E. coli* followed by infection of the target bacteriophage. The high efficiency of this technology, which yields 1–14% of desired recombinants, allows low-throughput screening for the desired mutant. We have demonstrated the use of this technology for single-base substitutions, for deletions of 50–201 bases, for insertions of 20 bases, and for four different phages. The technology may also be readily modified for use across many additional bacterial and phage strains.

**Figure:**
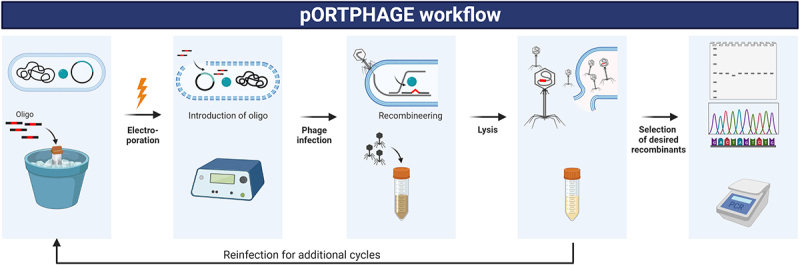

## Introduction

The abundance of phages and their importance to microbial evolution, potential therapeutic applications, and major ecological issues provide an incentive to study their biology [1]. Many homologous recombination-based methods to engineer phage genomes have been developed over the last two decades [2–6], recently reviewed by Brouns and colleagues [7]. One is the Bacteriophage Recombineering of Electroporated DNA (BRED) [2], in which the RecET recombination proteins are expressed in *Mycobacterium smegmatis*. These bacteria are then co-electroporated with a purified mycobacteriophage genome and a synthetic DNA substrate with a desired DNA modification. This procedure results in a high percentage of recombinants, enabling their identification using low-throughput PCR screens. Another method, occasionally called Bacteriophage Recombineering with Infectious Particles (BRIP) [3,4] uses λ lysogenic bacteria encoding the λ *red* genes in an integrated cryptic prophage. These lysogens are induced to express the λ Red recombination enzymes and are then electroporated with synthetic oligonucleotides that carry the desired DNA modification. The lysogens are then infected with the target phage (shown for λ or T7) and the high yield usually allows low throughput screening to identify the recombinants. A recent preprint describes another method for the genetic engineering of bacteriophages termed Recombitrons [6]. In contrast to previous methods, this method utilizes the most efficient recombination enzyme described to date, the CspRecT [8]. In this method, the recombination enzyme is induced in *E. coli*, and concomitantly, a plasmid encoded reverse transcriptase and a retron harbouring the desired modification are expressed, resulting in ssDNAs available for recombination. The *E. coli* also encodes a dominant-negative allele of the MutL protein, which temporarily silences the repair protein MutL and thus prevents repair of the introduced mutation [9]. The bacteria are then infected with the target phage, and plaques are isolated. The efficiency of this method is high, allowing the identification of recombinant phages by screening. The efficiencies and features of the above methods engineering coliphages are detailed in Supplementary Table S1. The major advantage of the above methods is that they do not require counter-selection techniques, such as employing CRISPR-Cas, for isolating the desired clones.

We were the first group to demonstrate the harnessing of the CRISPR-Cas system for counter-selecting recombinant phages [10] and have now been followed by many others [11–19]. Using CRISPR-Cas for genetic engineering is straightforward, can be tailored to virtually any gene, and is cost-effective. However, the counter-selection by CRISPR-Cas requires construction of specific plasmids for each genetic manipulation. In addition, it requires host cells encoding a functional CRISPR-Cas system (on plasmid\s or in the genome) and basic knowledge on CRISPR-Cas usage, which may refrain some labs from adopting it. We therefore believe that improving the methodologies that do not require counter-selection may become the method of choice for many labs requiring phage engineering.

Recently, new technologies that enable efficient engineering of many genomes, including bacterial genomes, have emerged, but these were not demonstrated for phage engineering [8,20]. One of these methods, pORTMAGE (Parallel Oligonucleotide Recombineering and Targeted Multiplex Genome Editing) enables the simultaneous and efficient modification of multiple sites in a genome by introducing a mutated oligonucleotide along with the inactivation of a critical repair protein, leading to homology-directed repair. Bacterial mutagenesis using pORTMAGE has proved successful with λ Red enzymes [20] and also subsequently with the significantly improved CspRecT recombination enzyme [8]. Although bacteriophage engineering may use similar principles, it requires adaptation of all the steps including protein induction, electrocompetent preparation, growth media, the timing of adding phages after electroporation of oligonucleotides, and multiplicity of infection.

Here, we describe a simple and efficient method, based on pORTMAGE, for genetic engineering of the *E. coli* phages T5, T7, P1, and λ. We term it pORTPHAGE and can use it to generate desired mutations at high efficiencies. The identification of the recombinants is carried out by simple Sanger DNA sequencing or PCR. We show by random sampling of the recombinants and by next-generation sequencing (NGS) that the efficiency of the technology enables low-throughput screening for the isolation of desired mutants from all tested phages. The technology allows the generation of point mutations, insertions, and deletions. Because the pORTMAGE plasmid is universally designed to function in multiple bacterial strains, only easily accomplished, minor modifications would be needed to make the method suitable for many other bacterial hosts.

## Materials and methods

### Reagents, strains, and plasmids

Lysogeny broth (LB) medium (1% w/v tryptone, 0.5% w/v yeast extract, 0.5% w/v NaCl) was obtained from Condalab, Terrific broth (TB) was from Sigma-Aldrich, Agar was from Hylabs. D-maltose, MgSO_4_, CaCl_2_, Kanamycin and M-Toluic acid were from Merck. KAPA HiFi HotStart Ready Mix was from Kapa Biosystems. PCRBIO HS Taq DNA polymerase was from PCR Biosystems. NucleoSpin Gel and PCR Clean-Up Kit were from Geneaid. All PCR standard oligodeoxynucleotides (oligos) were ordered with standard desalting from Merck. pORTPHAGE recombineering oligos containing two phosphorothioate (PT) bonds at each end (3’ and 5’, marked by asterisks) were ordered with standard desalting from Integrated DNA Technologies (IDT). Bacterial strains, plasmids, and oligonucleotides used in this study are listed in Table S2.

### Detailed pORTPHAGE protocol

A single colony from a strain harbouring the pORTMAGE-Ec1 plasmid was inoculated into LB medium supplemented with 50 μg/mL kanamycin and aerated in a shaking incubator at 37°C, 250 rpm for 16 h. Overnight (ON) cultures were diluted 1:100 in LB medium containing 50 µg/mL kanamycin and incubated at 37°C, 250 rpm. Upon reaching OD_600_ ~0.3, cells were incubated with 1 mM m-toluic acid for 30 min at 250 rpm to induce RecT expression. Immediately after induction, the cells were chilled on ice for at least 10 min and made electrocompetent by centrifugation at 4,800 × g for 10 min in a chilled (4°C) Thermo X4R Pro centrifuge with swing-out rotor and washing the pellet twice with 10 mL of ice-cold ddH_2_O. Electrocompetent cells were suspended in 160 μL ddH_2_O and were kept on ice until electroporation. Cell suspension (45 μL) was mixed with 5 μL of 100 μM oligonucleotide and electroporated in pre-chilled 2-mm gap electroporation cuvettes using a BioRad MicroPulser (1.8 kV, 200 Ω, 25 μF). Immediately after electroporation, 1 mL of room-temperature (RT) phage infection media was added, and the suspension was transferred to 4 mL RT media supplemented with phage lysate at an MOI of 1:1. Each phage was supplemented with different infection media. For T7, we used TB [20], for T5 we used LB supplemented with 10 mM MgSO_4_ +10 mM CaCl_2_ [5], for P1_vir_ we used LB supplemented with 5 mM CaCl_2_ [21] and for λ_vir_ we used LB supplemented with D-maltose 0.2% w/v and 10 mM MgSO_4_ [22]. Bacteria and phage mixture were incubated at 37°C under continuous agitation until the complete lysis of the culture. Chloroform (100 µL) was added to the lysates, which were mixed and centrifuged at 4,800 × g for 10 min, 4°C. The lysates were used for further rounds of infections as indicated. One aliquot (1 µL) was used as template for 20 cycles of PCR amplification using KAPA HiFi polymerase and primers containing different 8 nucleotide tags (marked as X) for each sample. Amplified products were purified using a PCR cleanup kit and subjected to NGS. The lysate was also serially diluted (10^−1^-10^−7^) and plated on a permitting host using the soft-agar overlay technique to prepare single plaque screening assays. The plates were incubated ON at 37°C (for T5, P1_vir_ and λ_vir_), or at RT (for T7). Single plaques were selected, suspended in 20 µL of LB, and used as template for PCR amplification using Taq polymerase and the same primers used for the NGS, but without the tags. Amplified products were purified and sequenced using the Sanger method with one of the primers used for amplification.

### NGS library preparation

The PCR products were purified and used as templates for a second PCR to barcode the samples specifically and enable NGS readings. The prepared Illumina sequencing libraries were sequenced by Novogene using a NovaSeq 6000 machine according to the manufacturer’s instructions, and a 2 × 150 flow cell. Samples were multiplexed in the same sequencing run. Demultiplexing was based on an 8-bp barcode that was part of the original PCR primer.

### NGS data analysis

The input is composed of paired-end sequencing. Each end is 150 bases long. The import programme looks for an overlap between the end of the forward read and the beginning of the reverse complement of the backward read and generates the combined sequence. Each generated sequence is grouped according to one of its given primers. The sequences are then divided into subgroups of equal length, and each nucleotide (A, C, G, T) is counted (=histogram) for each subgroup and location in the sequence. Gaps are counted as ‘Deleted’. In the other locations, the histograms are summed.

## Results

### The pORTPHAGE system

**Figure 1.**
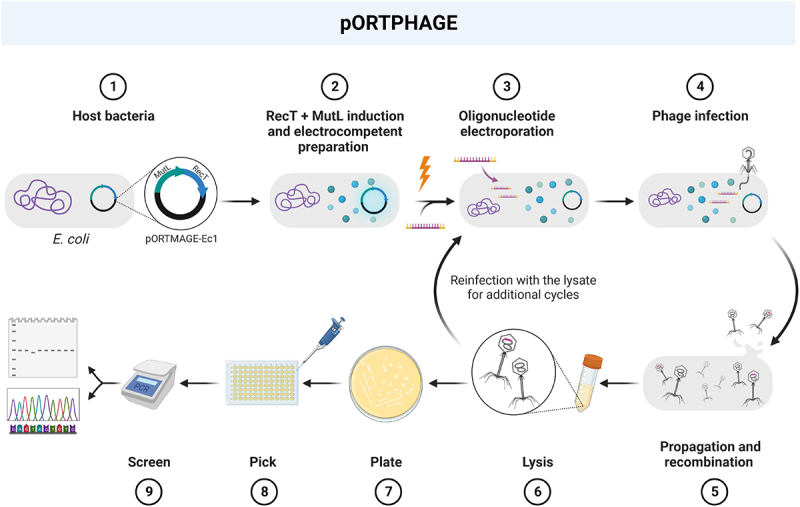
Schematic representation of the pORTPHAGE steps.

Evaluation of pORTPHAGE (a revised pORTMAGE method [20] adapted for phages) as an efficient selection-free and scarless technology for genetically engineering various phages, was conducted in *E. coli* as the host for the pORTPHAGE plasmid – pORTMAGE-Ec1 [8]. This plasmid encodes the recombination enzyme CspRecT, which possesses the highest reported recombination efficiency, and is derived from a phage infecting *Collinsella stercoris*. It also encodes a dominant-negative allele of the MutL protein, which temporarily silences the repair protein MutL and thus prevents repair of the introduced mutation [9]. We speculated that bacteria expressing these plasmid-encoded proteins will produce desired recombinant phages when infected with phages and electroporated with oligonucleotides encoding the mutations. We also speculated that the high efficiency of this procedure could allow affordable screening for the identification of the desired recombinants (Figure 1).

1. Transformation of pORTMAGE-Ec1 into the host bacteria. 2. Bacterial growth to early log phase followed by the expression of recombineering proteins and the preparation of electrocompetent bacteria. 3. Electroporation of oligonucleotides with desired mutation. 4. Infection of bacteria with the bacteriophage target. 5. Phage propagation and recombination. 6. Obtained lysate used for an additional cycle (step 3) or 7. Obtained lysate grown on bacterial host. 8. Single plaques are picked. 9. Relevant segments amplified by PCR and analysed on agarose gel or Sanger sequencing.

### Generation of point mutations in T7 phage

As the first step, we validated the ability to generate a point mutation in a T7 bacteriophage. For this purpose, we chose a T7 phage with an amber mutation in gene 1, which encodes T7-RNA polymerase, an essential protein for T7 phage propagation. This mutant phage can grow on an amber-suppressor host, such as NEB5α, but not on the wild-type *E. coli*. This phenotype provides an easy method to assess the recombination efficiency. We separately electroporated two oligonucleotides that encode a correction of the amber mutation in two reverse-complement orientations (which we arbitrarily term F and R) into an amber-suppressor host harbouring pORTMAGE-Ec1, and then infected it with the amber-mutated T7 phage. A similar procedure but with electroporation of water instead of oligonucleotides was used to produce the control. After two cycles, illustrated in Figure 1, we prepared plates with serial dilutions of the lysates, and replica-plated 96 plaques from each lysate on the *E. coli* and the NEB5α amber-suppressor host. We then examined the number of corrected phages obtained with the treated or untreated phages grown on *E. coli* or the amber suppressor host. A total of 11/96 corrected phages were obtained with one oligonucleotide, 4/96 with the complementary nucleotide, and 3/96 of spontaneous revertants in the untreated control sample. This result suggests that the pORTPHAGE mutates an average ~5% of the phage progeny after subtracting the background. Most treated recombinants (9/13) that grew on the wild-type host harboured the expected correction in Sanger sequencing. This procedure thus enables the isolation of desired mutations at high frequencies.

### Validation by NGS and by Sanger sequencing

To evaluate the mutagenesis efficiency by using an unbiased and statistically robust methodology, we carried out NGS on PCR-amplified segments from gene 1 encoding the mutation, which were grown on the amber-suppressor host without positive selection for the mutation. In accordance with the values described above, the NGS results indicated that the mutation occurred at a frequency of 4.49% for one oligonucleotide (F) and 7.87% for the other oligonucleotide (R) (Table 1). Moreover, the NGS showed that an additional cycle substantially increased the number of recombinants (Supplementary Table S4, and Supplementary Figure S1A).

**Table 1.** Point mutations obtained using pORTPHAGE in T7 and T5 phages.

Phage	Host	Targeted gene	Oligo orientation	Cycle	Sanger sequencing (%)	NGS (%)	Analysis – Table S3
T7	NEB5α	1	F	2	2.08	4.49	Tab 2
			R	2	7.29	7.87	Tab 4
		control	n.a.	2	n.d.	0.17	Tab 5
		17	F	2	0.00	2.01	Tab 7
			R	2	3.13	6.90	Tab 9
		control	n.a.	2	n.d.	0.03	Tab 10
T5	DH5α	D15	F	4	0.50	0.94	Tab 14
			R	4	n.d.	1.12	Tab 18
		control	n.a.	4	n.d.	0.05	Tab 19

### Point mutations in different phages

When we used the same protocol to mutate another T7 phage gene, namely gene 17, the desired mutation was detected by Sanger sequencing in 3.13% using one oligonucleotide (R), and 0.00% for the other orientation. The NGS analysis detected higher efficiencies of 6.90% and 2.01%, respectively (Table 1). This suggests that the orientation of the oligonucleotides affects the outcome [23], and that both should be employed in the procedure. Here too, the NGS showed that an additional cycle substantially increased the number of recombinants (Supplementary Table S4, Supplementary Figure S1A).

The protocol was slightly modified for mutating a T5 phage by completing four cycles before screening (Figure 1) due to the inconsistent efficiency of the mutagenesis of this phage [5]. Sanger screening yielded 0.50% of recombinants for one oligonucleotide (F) and 0.00% for the other (R), whereas the NGS results were 0.94% and 1.12% recombinants, respectively. Importantly, NGS detected negligible percentages of mutations in the no oligonucleotide controls (Table 1). The NGS showed that the additional cycles improved the efficiency in this case slightly after each cycle, consistent with the low efficiency of mutagenesis in the first cycle (Supplementary Table S4, Supplementary Figure S1A). These results demonstrate that we can use the protocols for easy screening for the desired recombinants without the need to establish the sort of negative selection required by CRISPR-Cas.

### Deletions in different phages

Encouraged by these results, we used similar protocols to delete 50 bp segments from T7, P1_vir_ and an entire gene (201bp) from λ_vir_. PCR can readily detect band shifts caused by deletions or insertions. Accordingly, the PCR analysis of individually isolated plaques revealed 5.00–6.25% recombinants in the T7 phage, 2.08–4.88% for P1_vir_ and 4.00–12.50% for λ_vir_. NGS detected even higher frequencies of mutagenesis in all analysed cases (Table 2). The λ_vir_ NGS could not be properly analysed since the sequenced region length size exceeded our run parameters. The NGS of the T7 deletion procedure showed a substantial increase with an additional cycle, but for P1 we observed an unexpected decrease for the additional cycle, suggesting that additional cycles may not always be required (Supplementary Table S5, and Supplementary Figure S1B). These results demonstrate that deletions can be obtained with high efficiency and can be readily detected using PCR.

**Table 2.** Deletions obtained by pORTPHAGE in T7, P1_vir_ and λ_vir_ phages.

Phage	Host	Targeted gene	Oligo orientation	Cycle	Sanger sequencing (%)	NGS (%)	Analysis – Table S3
T7	NEB5α	0.3	F	2	6.25	14.31	Tab 21
			R	2	5.00	4.82	Tab 23
		control*	n.a.	4	n.d.	0.00	Tab 24
P1_vir_	K-12	*pmgM*	F	2	4.88	5.57	Tab 26
			R	2	2.08	3.13	Tab 28
		control	n.a.	2	n.d.	0.30	Tab 29
λ_vir_	NEB5α	*ral*	F	2	12.50	n.d.	n.a.
			R	2	4.17	n.d.	n.a.
		control	n.a.	2	n.d.	n.d.	n.a.

*Same control used in Table 3.

### Insertion of 20 bp in the T7 phage

Finally, to demonstrate that the procedure is also effective for insertions, we used a similar protocol to insert 20 bp segments into the T7 phage, with oligonucleotides encoding the insertion. After four cycles as used previously, 4.17% recombinants with the desired insertion were detected by PCR for one oligonucleotide, with 0.00% for the other oligonucleotide. NGS analysis detected a 2.21% frequency for the first oligonucleotide and 1.02% for the other (Table 3). The NGS also showed substantial increase with each of the four cycles (Supplementary Table S6 and Supplementary Figure S1C). These results demonstrate that insertions can be obtained efficiently and can easily be screened using PCR.

**Table 3.** An insertion obtained by pORTPHAGE in the T7 phage.

Phage	Host	Targeted gene	Oligo orientation	Cycle	Sanger sequencing (%)	NGS (%)	Analysis – Table S3
T7	NEB5α	0.3	F	4	4.17	2.21	Tab 33
			R	4	0.00	1.02	Tab 37
		control	n.a.	4	n.d.	0.00	Tab 24

## Discussion

The technology for genetic engineering of phage genomes described here is adapted from a technology for bacterial genome engineering. Since phages cannot harbour the plasmid and rely on a bacterial host for propagation and recombination, the timing of the infection must be coordinated with induction of the recombination\repair silencing proteins and electroporation of the oligonucleotides. We now describe a system that synchronizes these events and provides robust mutagenesis efficiencies. A universal genetic engineering system to engineer phages should prove highly valuable for phage research and biotechnological use.

In Supplementary Table S1 we compare the procedures, efficiencies, and advantages and drawbacks of several phage engineering methods developed till now for coliphages. Of note is the Recombitron, which shows an overall highest efficiency among all of them. Nevertheless, we believe that the simplicity and requirements for less steps and reagents would render pORTPHAGE an appealing choice for labs interested in an efficient and reliable method for phage engineering. The use of the most efficient recombination enzyme described to date in combination with a dominant-negative MutL protein that disables the repair machinery provides mutagenesis frequencies of between 1% and 14%. These frequencies allow for easy screening of the desired mutants. The technology is easy to transfer and maintain in the lab – requiring only a bacterial host harbouring one plasmid and using commercially available 5’ and 3’-modified oligonucleotides. The mutagenesis procedure is straightforward, allowing the generation of several genetically engineered loci with less than a week of work. Indeed, we use the technology developed here routinely for engineering phages in our lab [24]. We believe that with a few adjustments, the system could easily be adapted to manipulate many phage genomes in many host strains.

## Supplementary Material

Supplemental Material

## Data Availability

The NGS data of the spacers generated in this study are available at the NCBI SRA with the BioProject accession – PRJNA975343.
