# Supplementary material for: An efficient, scarless, selection-free technology for phage engineering
Source: RNA Biol. 2023 Oct 16;20(1):830–5. doi: 10.1080/15476286.2023.2270344 (PMC10583621; doi:10.1080/15476286.2023.2270344)
Supplement: Supplemental Material [file KRNB_A_2270344_SM0265.zip › 230716 Supplementary information (3).docx]

**Supplementary Material**

**Supplementary Figure**

**A**

**B**

**
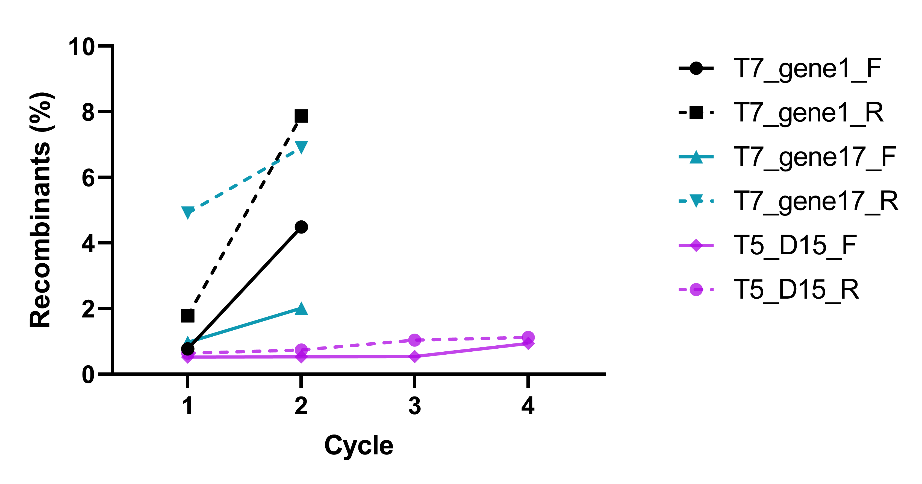
**
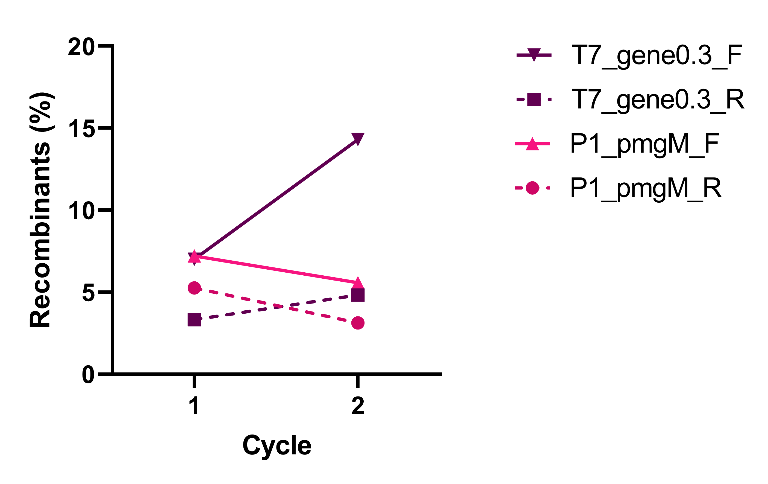


**
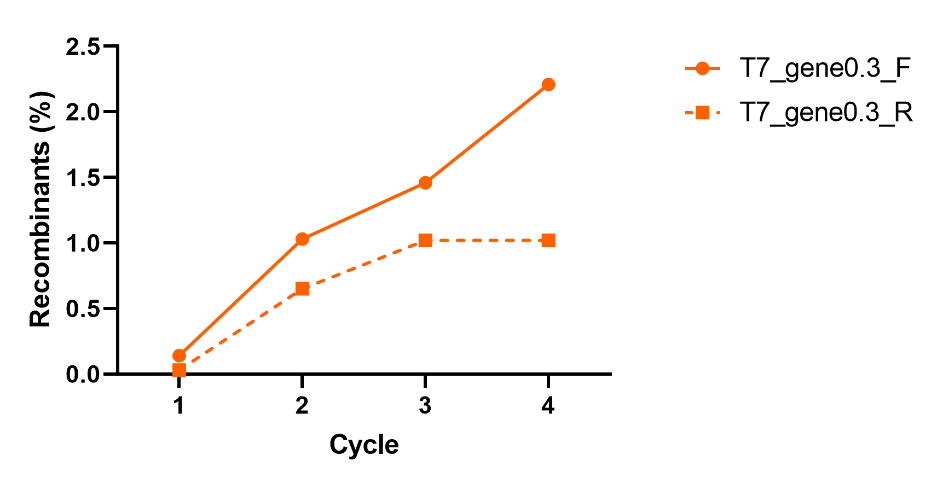
**

**C**

**Figure S1. The effect of additional cycles on recombinant’s percentage.** pORTPHAGE was carried out on T7, T5 and P1_vir_ phages, to produce: **A**. Point mutations, **B.** Deletions, and **C.** An insertion. The points on the graphs show the percentage of the recombinants identified by NGS at each cycle for achieving point mutations, as described in Materials and Methods. The names in the legend are formatted as: “Phage_Targeted gene_Oligo orientation”, as detailed in the columns of Tables S4, S5, S6.

**Supplementary Tables**

**Table S1. Comparison between pORTPHAGE and other non-CRISPR-based methods applicable for *E. coli strain***

|  | pORTPHAGE (this manuscript) | Lambda Red recombineering-based mutagenesis (BRIP) (1) | ReTRON-based mutagenesis (2) |
| --- | --- | --- | --- |
| Point mutation efficiency | T7 – 6.9%  T5 – 1.02%  2 cycles | λ – 1-3%  1 cycle | T7 – 1.5%  T5 – 0.6%  λ – 20%  1 cycle |
| Deletion efficiency | T7 – 4.82% (50bp)  P1 – 4.25% (50bp)  λ – 12.5% (201bp)  2 cycles | λ (326bp) – 2%  1 cycle | λ – 45% (32bp)  1 cycle |
| Insertion efficiency | T7 – 2.21% (20bp)  4 cycles | ND | λ – 10% (16bp), <1% (32bp)  1 cycle |
| Multiplexing efficiency | ND | ND | 93.2% in **1** or more sites after 3 cycles.  ~25% in 3 sites after 3 cycles |
| System components | - Phosphorothioated Oligonucleotide  - Single plasmid encoding both single-stranded annealing protein (SSAP), and MutL E32K | - Oligonucleotide  - Lysogen containing lambda prophage encoding Exo, Beta, and Gam | - One plasmid encoding Non-coding RNA (ncRNA), and Retron reverse transcriptase (RT)  - Second plasmid encoding SSAP, MutL E32K,  and Single-stranded binding protein (SSB) |
| Work process | - Designing mutagenizing oligonucleotides  - Inducing SSAP plasmid in the host  - Transformation of oligonucleotides  - Infecting with phage till lysis | - Designing mutagenizing oligonucleotides  - Inducing lysogen for expression of λ Red functions  - Transformation of oligonucleotides  - Inducing prophage excision till lysis | - Designing oligonucleotides for cloning  of mutagenizing ncRNA plasmid  - Cloning, extraction, and transformation of the mutagenizing plasmid  - Selection for two plasmids (retron and SSAP)  - Inducing plasmids in the host  - Infecting with phage till lysis |
| Summary | **Advantages**:  - No cloning required  - Wide host range for all types of mutations  - Best available recombinase\anti-repair system to date  - Phosphorothioated oligonucleotides reducing degradation  **Drawbacks**:  - Multiple oligonucleotide transformations | **Advantages**:  - No cloning required  **Drawbacks**:  - No disabling of the DNA repair system MutSHL  - λ Red expression from non-mobile lysogens  - Multiple oligonucleotide transformations | **Advantages**:  - Editing does not require oligonucleotide transformation  - Best available recombinase\anti-repair system to date  - High efficiency in most cases  **Drawbacks**:  - Phage SSB may inhibit the editing  - Anti retrons (e.g., in T5 phage) may inhibit efficiency (3)  - Narrower host range due to two plasmid system  - Requires genome editing to increase the mutagenesis rate for some mutations |

1. Oppenheim, A.B., Rattray, A.J., Bubunenko, M., Thomason, L.C. and Court, D.L. (2004) In vivo recombineering of bacteriophage lambda by PCR fragments and single-strand oligonucleotides. *Virology*, **319**, 185-189.

2. Fishman, C.B., Crawford, K.D., Bhattarai-Kline, S., Zhang, K., González-Delgado, A. and Shipman, S.L. (2023) Continuous Multiplexed Phage Genome Editing Using Recombitrons. *bioRxiv*, 2023.2003.2024.534024.

3. Azam, A.H., Chihara, K., Kondo, K., Nakamura, T., Ojima, S., Tamura, A., Yamashita, W., Cui, L., Takahashi, Y., Watashi, K. *et al.* (2023) Viruses encode tRNA and anti-retron to evade bacterial immunity. *bioRxiv*, 2023.2003.2015.532788.

**Table S2. Bacterial strains, plasmids and oligonucleotides used in this study.**

| **Bacterial strains** | **Description/sequence** | **Source or reference** |
| --- | --- | --- |
| *E. coli* NEB5α | F^-^ φ8*0lacZ*ΔM15Δ*(lacZYA-argF*) U169 *deoR re*c*A1* *endA1 hsdR17* (r_k_^-^, m_k_^+^) *gal ^-^ phoA supE44* λ^-^ *thi ^-^1 gyrA96 relA1* | New England Biolabs |
| *E. coli* DH5α | F^–^ *endA1 glnV44 thi-1 recA1 relA1 gyrA96 deoR nupG purB20* φ80d*lacZ*ΔM15 Δ(*lacZYA-argF*)U169, *hsdR17*(rK^–^mK^+^), λ^–^ | Thermo-Fisher scientific |
| *E. coli* K-12 MG1655 |  | HMS library |
| *E. coli* B |  | HMS library |
| **Bacteriophage strains** |  |  |
| T7 WT |  | Lab collection |
| T7 1am73 |  | Lab collection |
| T5 |  | Lab collection |
| P1_vir_ |  | Lab collection |
| λ_vir_ |  | Lab collection |
| **Plasmids** |  |  |
| pORTMAGE-Ec1 |  | Addgene |
|  |  |  |
| **Oligonucleotides** | **5'→3'** | **Used for** |
| MG420F | C*C*TACCATTAACACCAACAAAGATAGCGAGATTGATGCACACAAACAGGAGTCTGGTATCGCTCCTAACTTTGTACACAGCCAAGACG*G*T | T7 gene 1 point mutation |
| MG420R | T*A*CCGTCTTGGCTGTGTACAAAGTTAGGAGCGATACCAGACTCCTGTTTGTGTGCATCAATCTCGCTATCTTTGTTGGTGTTAATGGT*A*G | T7 gene 1 point mutation |
| MG440F | A*A*GACCATGAACCAGAACTCATGGCAAGCACGTAATGAAGCCTTATAGTTCCGTAATGAGGCTGAGACTTTCAGAAACCAAGCGGAGG*G*C | T7 gene 17 point mutation |
| MG440R | G*C*CCTCCGCTTGGTTTCTGAAAGTCTCAGCCTCATTACGGAACTATAAGGCTTCATTACGTGCTTGCCATGAGTTCTGGTTCATGGTC*T*T | T7 gene 17 point mutation |
| MG446F | T*G*ACTTACAACAACGTTTTCGACCACGCTTACGAAATGCTGAAAGATTCACATGGCTGCCGATAATGCAGTTCCGCACTACTACGCTG*A*C | T7 gene 0.3 50bp deletion |
| MG446R | G*T*CAGCGTAGTAGTGCGGAACTGCATTATCGGCAGCCATGTGAATCTTTCAGCATTTCGTAAGCGTGGTCGAAAACGTTGTTGTAAGT*C*A | T7 gene 0.3 50bp deletion |
| MG479F | T*G*ACTTACAACAACGTTTTCGACCACGCTTACGAAATGCTGAAAGTGACTGACTGACTGACTGACAAAACATCCGTTATGATGACATCCGTGACACTGATGACCTGCA*C*G | T7 gene 0.3 20bp insertion |
| MG479R | C*G*TGCAGGTCATCAGTGTCACGGATGTCATCATAACGGATGTTTTGTCAGTCAGTCAGTCAGTCACTTTCAGCATTTCGTAAGCGTGGTCGAAAACGTTGTTGTAAGT*C*A | T7 gene 0.3 20bp insertion |
| TM260F | A*A*AAGTACGCACAACGTACGGAAGAGGAGAAAGCGCTAGATGAGTAGTTCTTTGAGTATTTGAAGGATGCTTTCGAGTTGTGTAAAAC*T*A | T5 gene D15 point mutation |
| TM260R | T*A*GTTTTACACAACTCGAAAGCATCCTTCAAATACTCAAAGAACTACTCATCTAGCGCTTTCTCCTCTTCCGTACGTTGTGCGTACTT*T*T | T5 gene D15 point mutation |
| MG521F | A*A*ACAGCGTAAATATGGCATGCGACCGGCAGGATTTGACTGATTATGTTGGCGGTCCAGGCCAAAAACCAAAAAGGATCCATTCTTAT*C*G | P1_vir_ gene pmgM 50bp deletion |
| MG521R | C*G*ATAAGAATGGATCCTTTTTGGTTTTTGGCCTGGACCGCCAACATAATCAGTCAAATCCTGCCGGTCGCATGCCATATTTACGCTGT*T*T | P1_vir_ gene pmgM 50bp deletion |
| MG492F | G*T*GCAGTGTTTATTCTGTTATTTATGCCAAAAATAAAGGCCACTAACGGATAGTCCTGGTATTGTTCCATCACATCCTGAGGATGCTC*T*T | λ_vir_ gene ral 201bp deletion |
| MG492R | A*A*GAGCATCCTCAGGATGTGATGGAACAATACCAGGACTATCCGTTAGTGGCCTTTATTTTTGGCATAAATAACAGAATAAACACTGC*A*C | λ_vir_ gene ral 201bp deletion |
| MG457F | XXXXXXXXCCTGATGTTCCTCGGTCAGT | T7 gene 1 amplification |
| MG457R | XXXXXXXXGCCCACACTACAGTCTTACG | T7 gene 1 amplification |
| MG463F | XXXXXXXXTGCTCGTGGTCGTCGAATTG | T7 gene 17 amplification |
| MG463R | XXXXXXXXTTCGTCTCGGAAACCCTTGG | T7 gene 17 amplification |
| MG464F | XXXXXXXXACGAGGTAACACAAGATGGC | T7 gene 0.3 amplification |
| MG464R | XXXXXXXXTCGAACTCAAGGTCAATGCC | T7 gene 0.3 amplification |
| MG465F | XXXXXXXXCGTCTAGAACATCTACCAGAG | T5 gene D15 amplification |
| MG465R | XXXXXXXXTCGTCTGCTTCTACACCACG | T5 gene D15 amplification |
| MG502F | XXXXXXXXGGGAAGTGTCGTTACTGCAATGAATCG | P1vir gene pmgM amplification |
| MG526R | XXXXXXXXTTTGCCAGTGCTGCAAGTGC | P1vir gene pmgM amplification |
| MG495F | XXXXXXXXGTTACGAGCGACATTGCTCC | λvir gene ral amplification |
| MG495R | XXXXXXXXCCACCGAGCCTGATGTGGTT | λvir gene ral amplification |

**Table S3. Please see separate Excel sheet containing 37 tabs.**

**Table S4. Point mutations obtained using pORTPHAGE in T7 and T5 phages – data for all cycles.**

| Phage | Host | Targeted gene | Oligo orientation | Cycle | Sanger sequencing (%) | NGS (%) | Analysis - Table S3 |
| --- | --- | --- | --- | --- | --- | --- | --- |
| T7 | NEB5α | 1 | F | 1 | n.d. | 0.77 | Tab 1 |
|  |  |  |  | 2 | 2.08 | 4.49 | Tab 2 |
|  |  |  | R | 1 | n.d. | 1.78 | Tab 3 |
|  |  |  |  | 2 | 7.29 | 7.87 | Tab 4 |
|  |  | control | n.a. | 2 | n.d. | 0.17 | Tab 5 |
|  |  | 17 | F | 1 | n.d. | 0.97 | Tab 6 |
|  |  |  |  | 2 | 0 | 2.01 | Tab 7 |
|  |  |  | R | 1 | n.d. | 4.91 | Tab 8 |
|  |  |  |  | 2 | 3.13 | 6.90 | Tab 9 |
|  |  | control | n.a. | 2 | n.d. | 0.03 | Tab 10 |
| T5 | DH5α | D15 | F | 1 | n.d. | 0.52 | Tab 11 |
|  |  |  |  | 2 | n.d. | 0.53 | Tab 12 |
|  |  |  |  | 3 | n.d. | 0.54 | Tab 13 |
|  |  |  |  | 4 | 0.5 | 0.94 | Tab 14 |
|  |  |  | R | 1 | n.d. | 0.64 | Tab 15 |
|  |  |  |  | 2 | n.d. | 0.74 | Tab 16 |
|  |  |  |  | 3 | n.d. | 1.04 | Tab 17 |
|  |  |  |  | 4 | n.d. | 1.12 | Tab 18 |
|  |  | control | n.a. | 4 | n.d. | 0.05 | Tab 19 |

**Table S5. Deletions obtained by pORTPHAGE in T7, P1_vir_ and** λ_vir_ **phages – data for all cycles.**

| Phage | Host | Targeted gene | Oligo orientation | Cycle | Sanger sequencing (%) | NGS (%) | Analysis - Table S3 |
| --- | --- | --- | --- | --- | --- | --- | --- |
| T7 | NEB5α | 0.3 | F | 1 | n.d. | 7.00 | Tab 20 |
|  |  |  |  | 2 | 6.25 | 14.31 | Tab 21 |
|  |  |  | R | 1 | n.d. | 3.32 | Tab 22 |
|  |  |  |  | 2 | 5.00 | 4.82 | Tab 23 |
|  |  | control | n.a. | 4 | n.d. | 0.00 | Tab 24 |
| P1_vir_ | K-12 | *pmgM* | F | 1 | n.d. | 7.21 | Tab 25 |
|  |  |  |  | 2 | 4.88 | 5.57 | Tab 26 |
|  |  |  | R | 1 | n.d. | 5.26 | Tab 27 |
|  |  |  |  | 2 | 2.08 | 3.13 | Tab 28 |
|  |  | control | n.a. | 2 | n.d. | 0.30 | Tab 29 |
| λ_vir_ | NEB5α | *ral* | F | 1 | n.d. | n.d. | n.a. |
|  |  |  |  | 2 | 12.50 | n.d. | n.a. |
|  |  |  | R | 1 | n.d. | n.d. | n.a |
|  |  |  |  | 2 | 4.00 | n.d. | n.a. |
|  |  | control | n.a. | 2 | n.d. | n.d. | n.a. |

| Phage | Host | Targeted gene | Oligo orientation | Cycle | Sanger sequencing (%) | NGS (%) | Analysis - Table S3 |
| --- | --- | --- | --- | --- | --- | --- | --- |
| T7 | NEB5α | 0.3 | F | 1 | n.d. | 0.14 | Tab 30 |
|  |  |  |  | 2 | n.d. | 1.03 | Tab 31 |
|  |  |  |  | 3 | n.d. | 1.46 | Tab 32 |
|  |  |  |  | 4 | 4.17 | 2.21 | Tab 33 |
|  |  |  | R | 1 | n.d. | 0.03 | Tab 34 |
|  |  |  |  | 2 | n.d. | 0.65 | Tab 35 |
|  |  |  |  | 3 | n.d. | 1.02 | Tab 36 |
|  |  |  |  | 4 | 0.00 | 1.02 | Tab 37 |
|  |  | control | n.a. | 4 | n.d. | 0.00 | Tab 24 |

**Table S6. An insertion obtained by pORTPHAGE in the T7 phage – data for all cycles.**
